# Supplementary material for: Development of Neutralization Breadth against Diverse HIV‐1 by Increasing Ab–Ag Interface on V2
Source: Adv Sci (Weinh). 2022 Mar 23;9(15):2200063. doi: 10.1002/advs.202200063 (PMC9130890; doi:10.1002/advs.202200063)
Supplement: Supplementary file 1 — Supporting Information [file ADVS-9-2200063-s001.pdf]

ADVANCED SCIENCE
Open Access

Supporting Information

for *Adv. Sci.*, DOI 10.1002/advs.202200063

Development of Neutralization Breadth against Diverse HIV-1 by Increasing Ab–Ag Interface on V2

Nan Gao, Yanxin Gai, Lina Meng, Chu Wang, Wei Wang, Xiaojun Li, Tiejun Gu, Mark K. Louder, Nicole A. Doria-Rose, Kevin Wiehe, Alexandra F. Nazzari, Adam S. Olia, Jason Gorman, Reda Rawi, Wenmin Wu, Clayton Smith, Htet Khant, Natalia de Val, Bin Yu, Junhong Luo, Haitao Niu, Yaroslav Tsybovsky, Huaxin Liao, Thomas B. Kepler, Peter D. Kwong, John R. Mascola, Chuan Qin, Tongqing Zhou*, Xianghui Yu* and Feng Gao**

Supporting Information

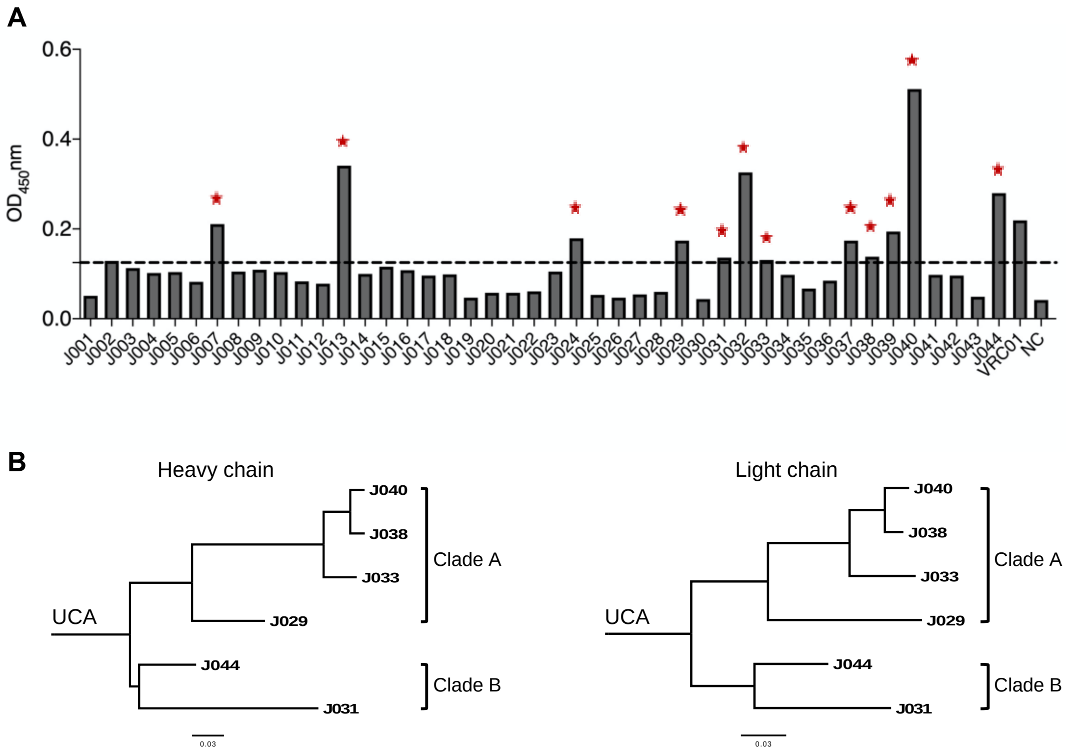

Supplementary Figure 1. Binding of newly isolated mAbs to autologous gp120 and Maximum-likelihood phylogenetic tree of the J038 lineage antibodies. (A) Supernatants from the 293T cells transfected with paired antibody heavy/light chain linear DNA fragments (mAb ID indicated on the x-axis) were assayed by ELISA for binding to autologous Env (gp120). The mAbs that bind to gp120 are indicated by red stars. Supernatant from 293T cells transfected with VRC01 and from mock-transfection were used as positive control and negative controls (NC). The cutoff (2.1-fold of the NC value) for positive binding is indicated by the dash line. (B) Maximum-likelihood phylogenetic tree of the heavy and light chain sequences of the J038 lineage antibodies. Unmutated common ancestor (UCA) of the six broadly neutralizing antibodies from the same gene family was inferred.Type or paste caption here. Create a page break and paste in the Figure above the caption.

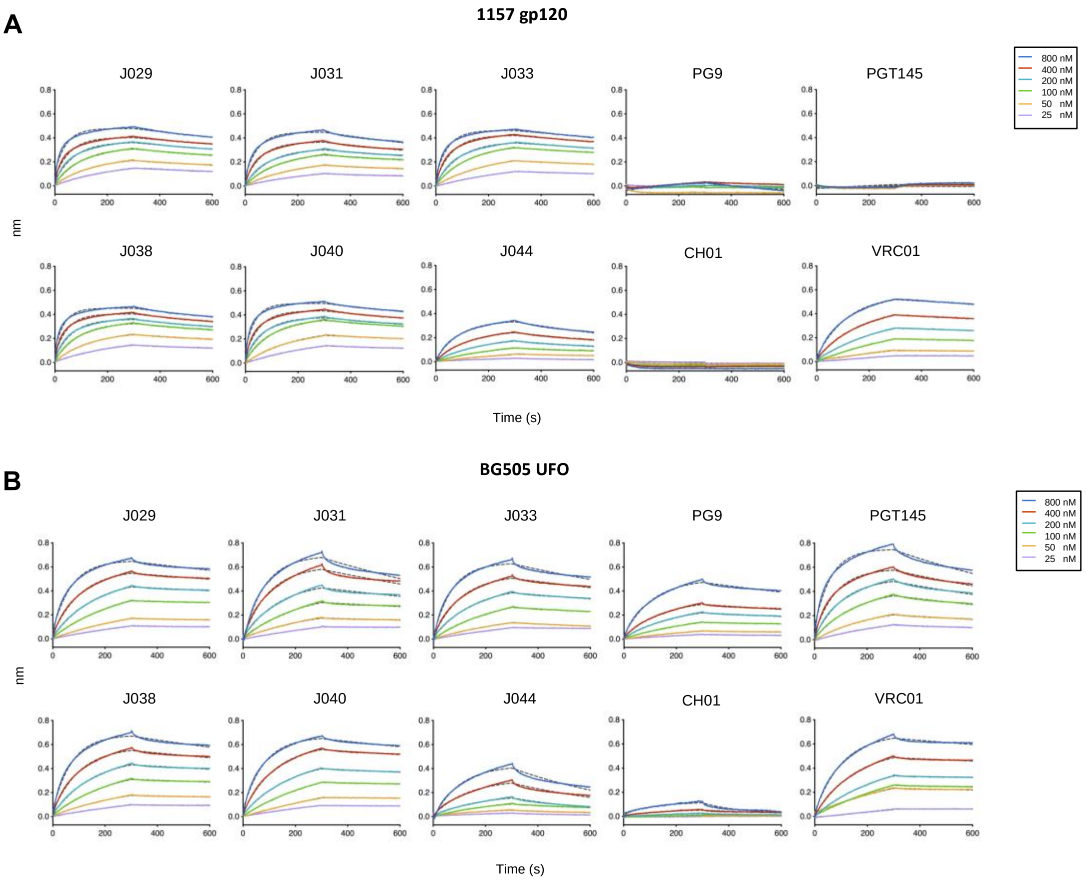

C

Antigen		J029	J031	J033	J038	J040	J044	PG9	PGT145	CH01	VRC01
**1157 gp120**	Kon (1/Ms)	6.57E+04	4.80E+04	4.34E+04	7.95E+04	6.75E+04	1.55E+04	N/A	N/A	N/A	1.29E+04
	Kdis (1/s)	5.46E-04	4.57E-04	6.92E-04	6.01E-04	4.92E-04	1.08E-03	N/A	N/A	N/A	2.77E-04
	KD (nM)	8.3	9.5	15.9	7.6	7.3	69.5	N/A	N/A	N/A	21.4
**BG505 trimer**	Kon (1/Ms)	2.31E+04	1.72E+04	2.04E+04	2.26E+04	1.96E+04	1.01E+04	1.32E+04	3.01E+04	1.31E+04	1.35E+04
	Kdis (1/s)	3.45E-04	7.57E-04	6.76E-04	4.41E-04	3.06E-04	2.03E-03	6.06E-04	9.33E-04	3.44E-03	2.73E-04
	KD (nM)	15.0	44.0	33.1	19.5	15.6	201.0	45.8	31.0	263.0	20.2

Supplementary Figure 2. Binding affinity of the J038 lineage antibodies. (A) The binding kinetics of six J038 lineage antibodies to autologous SHIV$_{1157ipd3N4}$ monomer gp120 were determined by BLI. The colored lines are the experimental curves for association and dissociation of the binding events, and the fitting curves were shown with gray dashed lines. (B) The binding kinetics of six J038 lineage antibodies to autologous BG505 trimer were determined by BLI. The colored lines are the experimental curves for association and dissociation of the binding events, and the fitting curves were shown with gray dashed lines. (C) Affinity constants for the J038 lineage antibodies. N/A; not applicable. Type or paste caption here. Create a page break and paste in the Figure above the caption.

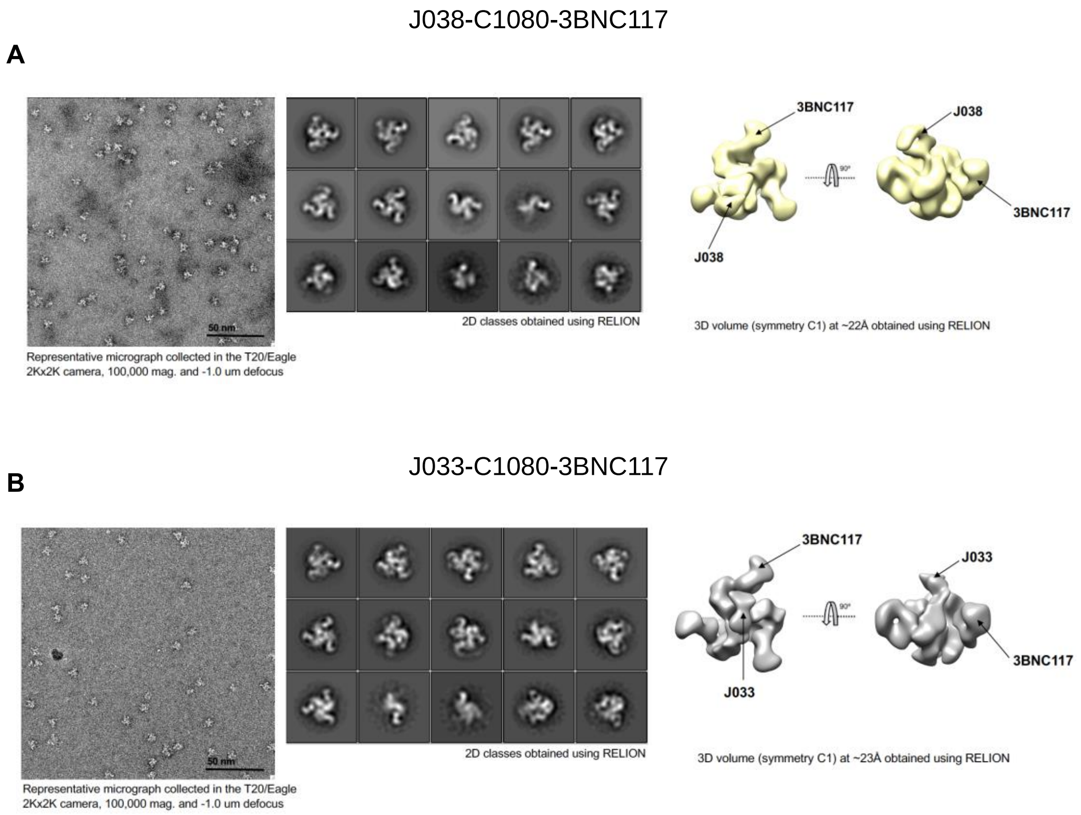

Supplementary Figure 3. Negative-stain EM and Cryo-EM of antibody-Env complex. (A) Representative reference-free 2D class averages of J038-C1080 Env-3BNC117 complexes were shown at the middle panel. (B) Representative reference-free 2D class averages of J033-C1080 Env-3BNC117 complexes were shown at the middle panel. Type or paste caption here.

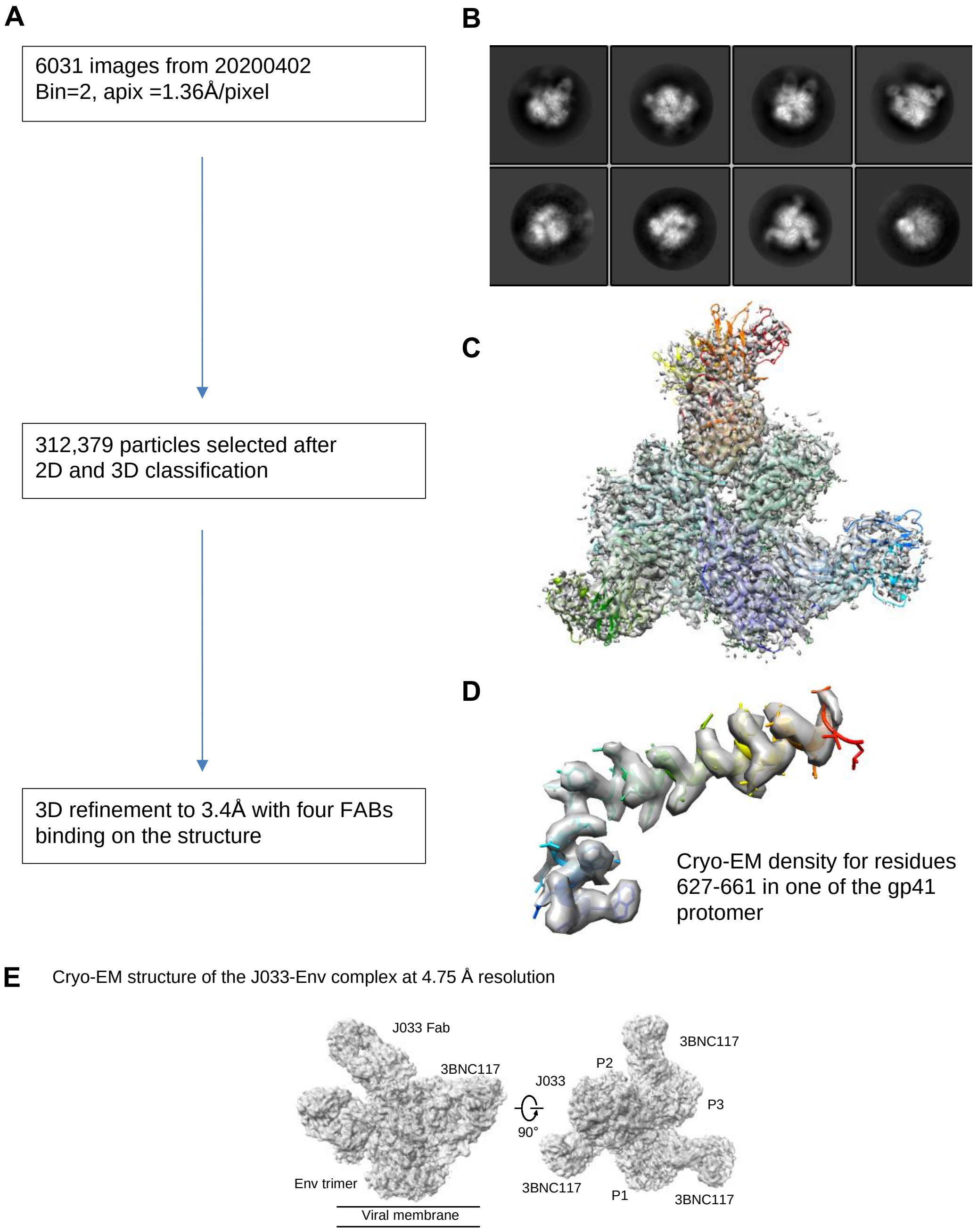

Supplementary Figure 4. Cryo-EM details of J038 in complex with HIV-1 Env. (A) Work follows of image processing. (B) 2D classes. (C) Model fitting into the map, colored by chains. (D) Representation region of the cryo-EM density with fitted residues. (E) Cryo-EM structure of the Fab J033-Env complex, with EM reconstruction density shown in gray. The CD4-binding site antibody 3BNC117 was used to aid the resolution. Protomers of the trimer are labeled as P1, P2 and P3.

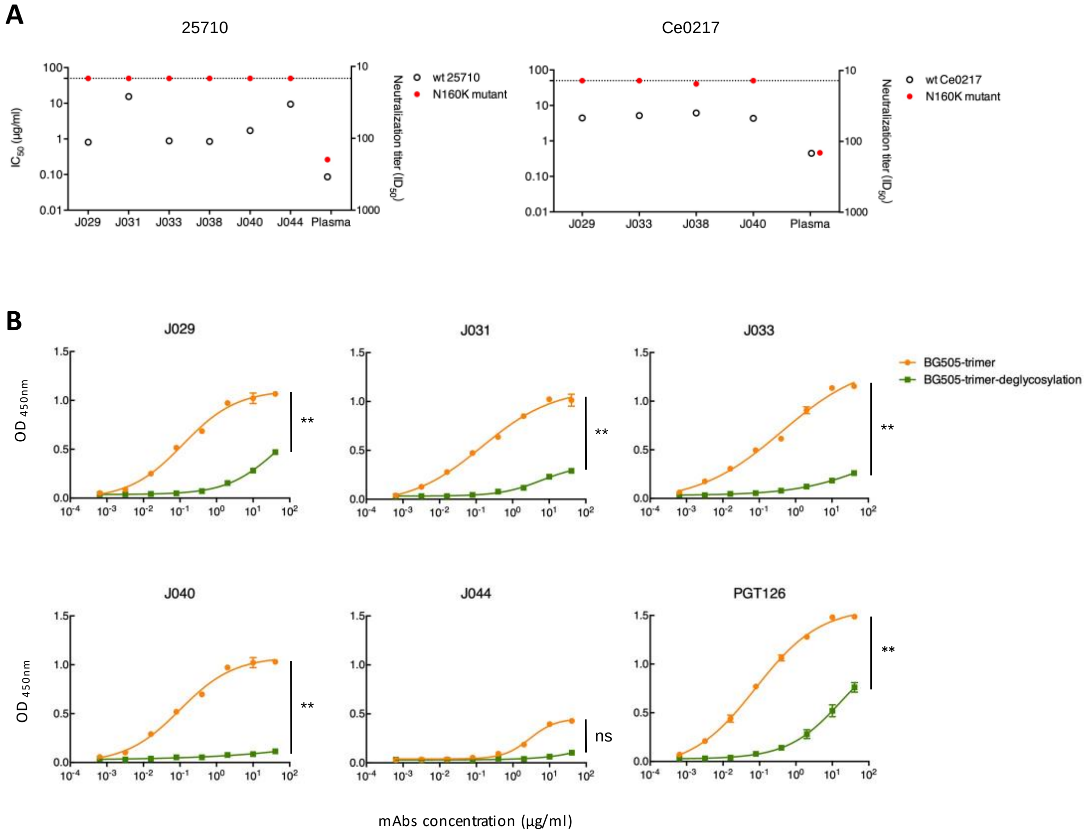

Supplementary Figure 5: The influence of glycan on antigen recognition of the J038 lineage antibodies. (A) The N160K mutation rendered heterologous viruses 25710 and Ce0217 highly resistant to neutralization by the J038 lineage antibodies. (B) The binding to the deglycosylated BG505 trimer by the J038 lineage antibodies was dramatically reduced. PGT126 serves as a positive control. Paired *t*-test was used for statistical analysis of 8-seiral dilution ELISA binding data. Data were presented with mean ± SD. **: $p < 0.01$; *: $p < 0.05$; ns: not significant.

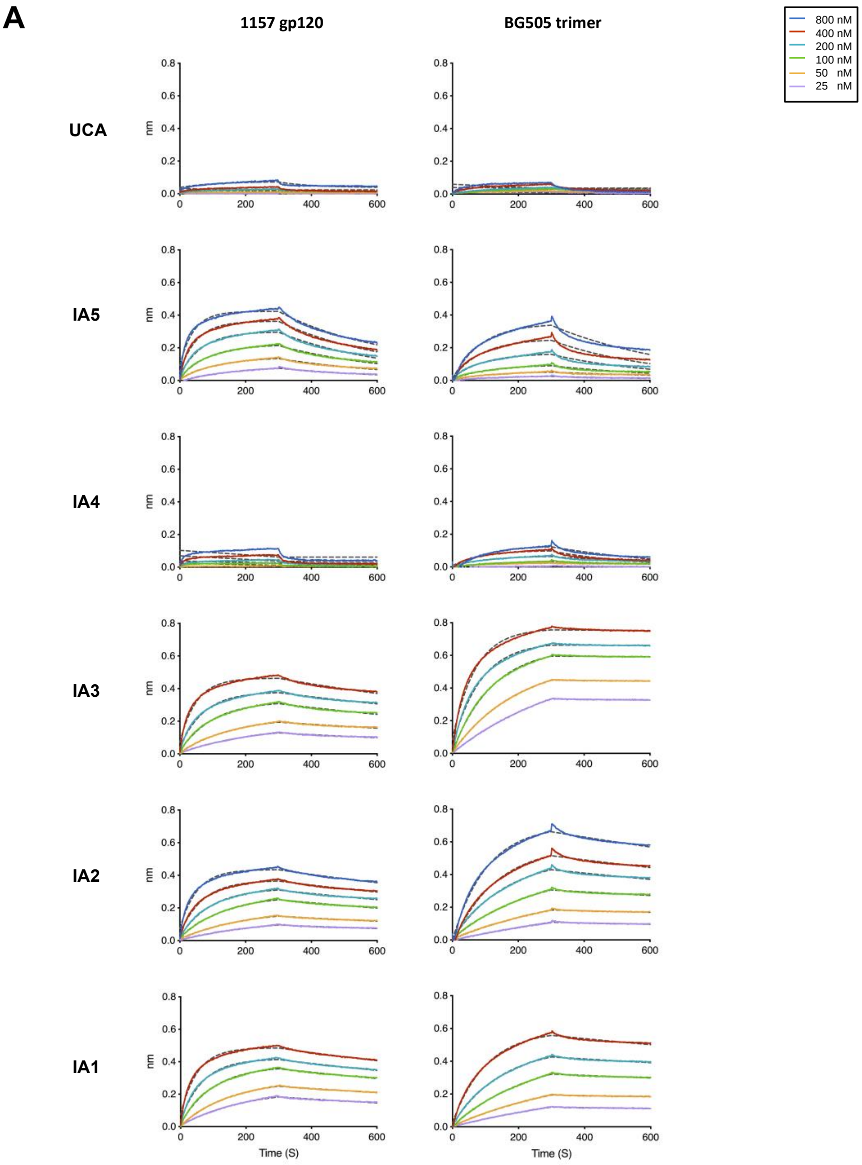

B

Antigen		UCA	IA5	IA4	IA3	IA2	IA1
**1157 gp120**	Kon (1/Ms)	6.09E+04	5.87E+04	1.38E+05	7.43E+04	4.18E+04	7.86E+04
	Kdis (1/s)	2.30E-03	2.41E-03	4.27E-03	7.42E-04	7.27E-04	6.17E-04
	KD (nM)	37.8	41.0	30.9	10.0	17.4	7.8
**BG505 trimer**	Kon (1/Ms)	3.66E+04	1.40E+04	7.92E+03	5.69E+04	1.25E+04	2.77E+04
	Kdis (1/s)	4.26E-03	2.84E-03	3.46E-03	6.17E-05	6.00E-04	3.43E-04
	KD (nM)	116.4	202.9	436.9	1.1	47.9	12.4

Supplementary Figure 6. Binding affinity of inferred UCA and IAs of the J038 lineage antibodies. (A) The binding kinetics of UCA and IAs of the J038 lineage antibodies to the autologous $SHIV_{1157ipd3N4}$ monomer gp120 or the BG505 trimer were determined by BLI. The colored lines are the experimental curves for association and dissociation of the binding events, and the fitting curves were shown with gray dashed lines. (B) Affinity constants of the UCA and IAs of the J038 lineage antibodies.

Supplementary Table 1. Lineage analysis and sequence characteristics of 12 monoclonal antibodies isolated from G1015R.

	Heavy chain					Light chain			
mAb ID	IGHV	IGHD	IGHJ	CDR3 (aa)	SHM (%)	IGKV/LV	IGKJ/LJ	CDR3 (aa)	SHM (%)
J029	4-j*02	3-9*01	5-1*01	AREVAEDDFGYYQPYYDS	18	K1-q*02	K4*01	QQYKALPLT	20
J031	4-j*02	3-9*01	4*01	AREVPVDDYGYYLPYFDP	22	K1-b*06	K4*01	QQYVSMPLT	17
J033	4-j*02	3-9*01	4*01	AGETPEDDFGYYQPYFKS	23	K1-q*02	K4*01	QHYKRLPLT	20
J038	4-j*02	3-9*01	4*01	AGETPEDDFGYYQPYFKT	22	K1-q*02	K4*01	QHYRRLPLT	16
J040	4-j*02	3-9*01	4*01	AGETPEDDFGYYQPYFKS	23	K1-q*02	K4*01	QHYKRLPLT	15
J044	4-j*02	3-9*01	4*01	AREVAVDEYNYYAPYFDS	11	K1-b*06	K4*01	QQYKSLPLT	14
J039	4-n*01	4-4*02	4*01	ATQSPLDGMSFGLNVA	11	K3-e*05	K1*01	HQYSDSVPWT	11
J024	4-e*01	5-5*02	5-1*01	ARRRGDWLLSTKRTWFDV	13	K1-q*04	K1*01	QQGYNYPRT	9
J013	3-y*04	2-25*01	5-1*01	IVGTKHCGDLYCPVAWFDV	7	K3-f*02	K2*01	QKQRDSPYT	11
J037	3-r*03	3-26*02	5-1*01	VRGSHRGDYNRFFRGPKTSFDL	19	K1-r*01	K2*01	QHYYRSPYS	6
J032	3-r*03	3-21*01	5-1*01	VRASHRGNYDRFFFSQKTWFDV	10	K3-f*02	K2*01	QEYDSYPYS	6
J007	3-r*03	3-26*02	5-1*01	VRGSHRGDYDRFFRSPKTSFDL	16	L8-a*01	L6*01	TLYMGSGISM	2

Supplementary Table 2. Neutralization activity of J038 against a panel of 208 viruses.

Virus	Clade	IC_{50} (μg/ml)	IC_{80} (μg/ml)
0260.v5.c36	A	>50	>50
0330.v4.c3	A	4.25	16.2
0439.v5.c1	A	>50	>50
3365.v2.c20	A	2.53	10.1
3415.v1.c1	A	>50	>50
3718.v3.c11	A	12.6	34.1
398-F1.F6.20	A	8.24	48.5
BB201.B42	A	1.54	6.09
BB539.2B13	A	6.00	22.4
BG505.W6M.C2	A	8.67	29.5
BI369.9A	A	13.4	45.2
BS208.B1	A	0.833	4.90
KER2008.12	A	1.01	2.99
KER2018.11	A	1.71	4.21
KNH1209.18	A	>50	>50
MB201.A1	A	>50	>50
MB539.2B7	A	27.3	>50
MI369.A5	A	>50	>50
MS208.A1	A	38.3	>50
Q23.17	A	9.06	>50
Q259.17	A	11.4	>50
Q769.d22	A	>50	>50
Q769.h5	A	>50	>50
Q842.d12	A	6.53	30.2
QH209.14M.A2	A	>50	>50
RW020.2	A	>50	>50
UG037.8	A	3.25	8.33
246-F3.C10.2	AC	30.3	>50
3301.v1.c24	AC	7.35	35.6
3589.v1.c4	AC	2.39	9.50
6540.v4.c1	AC	5.51	16.2
6545.v4.c1	AC	5.05	15.6
0815.v3.c3	ACD	>50	>50
6095.v1.c10	ACD	1.23	39.2
3468.v1.c12	AD	>50	>50
Q168.a2	AD	12.6	34.7
Q461.e2	AD	>50	>50
620345.c1	AE	>50	>50
BJOX009000.02.4	AE	35.0	>50
BJOX010000.06.2	AE	25.1	>50
BJOX025000.01.1	AE	>50	>50
BJOX028000.10.3	AE	>50	>50
C1080.c3	AE	0.095	0.305
C2101.c1	AE	4.27	11.5
C3347.c11	AE	>50	>50
C4118.09	AE	1.81	4.71
CM244.ec1	AE	1.07	2.99
CNE3	AE	>50	>50
CNE5	AE	1.73	4.45
CNE55	AE	14.2	>50
CNE56	AE	>50	>50
CNE59	AE	>50	>50
CNE8	AE	>50	>50
M02138	AE	>50	>50
R1166.c1	AE	>50	>50
R2184.c4	AE	>50	>50
R3265.c6	AE	>50	>50
TH023.6	AE	0.009	0.067
TH966.8	AE	1.36	4.59
TH976.17	AE	>50	>50
235-47	AG	>50	>50
242-14	AG	6.23	>50
263-8	AG	17.5	>50
269-12	AG	49.9	>50
271-11	AG	>50	>50
928-28	AG	6.48	42.1
DJ263.8	AG	0.054	0.194
T250-4	AG	0.839	2.79
T251-18	AG	>50	>50
T253-11	AG	>50	>50

Virus	Clade	IC_{50} (μg/ml)	IC_{80} (μg/ml)
T255-34	AG	>50	>50
T257-31	AG	11.5	28.6
T266-60	AG	20.6	>50
T278-50	AG	12.3	>50
T280-5	AG	17.8	42.1
T33-7	AG	>50	>50
3988.25	B	4.23	15.2
5768.04	B	14.2	>50
6101.1	B	>50	>50
6535.3	B	>50	>50
7165.18	B	28.9	>50
45_01dG5	B	7.04	27.4
89.6.DG	B	>50	>50
AC10.29	B	8.73	32.1
ADA.DG	B	0.347	1.64
Bal.01	B	0.118	0.319
BaL.26	B	0.163	0.545
BG1168.01	B	>50	>50
BL01.DG	B	>50	>50
BR07.DG	B	>50	>50
BX08.16	B	0.087	0.341
CAAN.A2	B	>50	>50
CNE10	B	>50	>50
CNE12	B	>50	>50
CNE14	B	>50	>50
CNE4	B	>50	>50
CNE57	B	>50	>50
HO86.8	B	1.95	5.96
HT593.1	B	3.79	15.0
HXB2.DG	B	0.010	0.032
JRCSF.JB	B	0.144	0.596
JRFL.JB	B	>50	>50
MN.3	B	0.393	17.9
PVO.04	B	>50	>50
QH0515.01	B	>50	>50
QH0692.42	B	>50	>50
REJO.67	B	0.641	1.63
RHPA.7	B	12.2	>50
SC422.8	B	13.8	>50
SF162.LS	B	>50	>50
SS1196.01	B	0.641	1.97
THRO.18	B	3.82	16.6
TRJO.58	B	>50	>50
TRO.11	B	14.6	>50
WITO.33	B	0.334	1.58
X2278.C2.B6	B	0.572	1.82
YU2.DG	B	8.99	24.5
BJOX002000.03.2	BC	2.71	43.2
CH038.12	BC	>50	>50
CH070.1	BC	1.95	4.70
CH117.4	BC	1.23	5.68
CH119.10	BC	47.2	>50
CH181.12	BC	>50	>50
CNE15	BC	5.83	22.2
CNE19	BC	4.15	18.6
CNE20	BC	>50	>50
CNE21	BC	>50	>50
CNE40	BC	0.231	>50
CNE7	BC	>50	>50
286.36	C	10.8	33.9
288.38	C	4.11	20.1
0013095-2.11	C	1.55	3.67
001428-2.42	C	2.02	4.93
0077.v1.c16	C	5.25	28.5
00836-2.5	C	>50	>50
0921.v2.c14	C	1.73	5.95
16055-2.3	C	2.25	5.89
16845-2.22	C	>50	>50
16936-2.21	C	>50	>50
25710-2.43	C	1.64	6.60

Virus	Clade	IC_{50} (μg/ml)	IC_{80} (μg/ml)
25711-2.4	C	>50	>50
25925-2.22	C	>50	>50
26191-2.48	C	>50	>50
3168.v4.c10	C	2.33	7.85
3637.v5.c3	C	>50	>50
3873.v1.c24	C	43.0	>50
426c	C	>50	>50
6322.v4.c1	C	>50	>50
6471.v1.c16	C	>50	>50
6631.v3.c10	C	>50	>50
6644.v2.c33	C	0.039	0.124
6785.v5.c14	C	1.01	3.50
6838.v1.c35	C	3.40	13.7
96ZM651.02	C	>50	>50
BR025.9	C	0.480	3.30
CAP210.E8	C	0.932	9.38
CAP244.D3	C	>50	>50
CAP256.206.C9	C	10.6	35.2
CAP45.G3	C	0.259	1.02
Ce1176.A3	C	>50	>50
CE703010217.B6	C	1.54	4.66
CNE30	C	>50	>50
CNE31	C	>50	>50
CNE53	C	>50	>50
CNE58	C	2.66	10.6
DU123.06	C	1.31	4.34
DU151.02	C	2.03	6.05
DU156.12	C	18.8	>50
DU172.17	C	13.2	44.0
DU422.01	C	>50	>50
MW965.26	C	0.094	0.790
SO18.18	C	19.5	>50
TV1.29	C	0.889	3.86
TZA125.17	C	30.7	>50
TZBD.02	C	>50	>50
ZA012.29	C	>50	>50
ZM106.9	C	>50	>50
ZM109.4	C	1.00	7.06
ZM135.10a	C	>50	>50
ZM176.66	C	0.677	2.46
ZM197.7	C	>50	>50
ZM214.15	C	>50	>50
ZM215.8	C	>50	>50
ZM233.6	C	0.096	0.459
ZM249.1	C	22.8	>50
ZM53.12	C	>50	>50
ZM55.28a	C	>50	>50
3326.v4.c3	CD	>50	>50
3337.v2.c6	CD	>50	>50
3817.v2.c59	CD	12.5	>50
191821.E6.1	D	>50	>50
231965.c01	D	14.6	>50
247-23	D	>50	>50
3016.v5.c45	D	>50	>50
57128.vrc15	D	>50	>50
6405.v4.c34	D	>50	>50
A03349M1.vrc4a	D	>50	>50
A07412M1.vrc12	D	>50	>50
NKU3006.ec1	D	>50	>50
UG021.16	D	>50	>50
UG024.2	D	0.159	0.629
P0402.c2.11	G	>50	>50
P1981.C5.3	G	16.4	>50
X1193.c1	G	9.88	21.6
X1254.c3	G	>50	>50
X1632.S2.B10	G	12.0	>50
X2088.c9	G	>50	>50
X2131.C1.B5	G	0.898	2.42
SIVmac251.30.SG3	NA	>50	>50
SVA.MLV	NA	>50	>50

<0.001	0.001-0.01	0.01-0.100	0.100-1.00	1.00-10.0	>10.0

Neutralization potency was shown as IC_{50} and IC_{80} and color coded as indicated.

Supplementary Table 3. Cryo-EM data collection, refinement and validation statistics for J038 and J033 in complex with HIV-1 Env.

	C1080 Env in complex with J038 and 3BNC117 (EMDB:EMD-24071) (PDB:7MXD)	C1080 Env in complex with J033 and 3BNC117 (EMDB:EMD-24128) (PDB:7N28)
Data collection and processing		
Magnification	18,000 Super-res	18000 Super-res
Voltage (kV)	300	300
Electron exposure (e^-/Å^2)	50.0	50.0
Defocus range (μm)	-1.6 to -3.0	-1.6 to -3.0
Pixel size (Å)	0.68/1.36(bin=2)	0.68/1.02 (bin=1.5)
Symmetry imposed	C1	C1
Final particle images (no.)	312,379	383,584
Map resolution (Å)	3.40	4.20
FSC threshold	0.143	0.143
Refinement		
Initial model used (PDB code)		
Model resolution (Å)	3.64	
FSC threshold	0.143	0.143
Map sharpening B factor (Å^2)	-89.5	-129.8
Model composition		
Non-hydrogen atoms	29496	29455
Protein residues	3519	3522
Ligands	145	140
B factors (Å^2)(mean)		
Protein	186	88
Ligand	189	103
R.m.s. deviations		
Bond lengths (Å)	0.003	0.003
Bond angles (°)	0.640	0.583
Validation		
MolProbity score	1.73	1.97
Clash score	6.6	10.2
Poor rotamers (%)	0.58	0.03
Ramachandran plot		
Favored (%)	94.7	93.0
Allowed	5.3	7.0
Disallowed	0	0

Supplementary Table 4. Interaction between J038 and HIV-1 Env.

A. Interface areas of paratope and epitope

Antibody	HIV	Type	Epitope (Å^2)		Paratope (Å^2)	
Heavy chain	Protomer P1	Protein	418		409	
		Glycan 156				
		NAG717	28		25	
		NAG718	73		70	
		Glycan 160				
		NAG770	86		75	
		NAG771	124		99	
		BMA772	61		62	
		MAN776	131		99	
		MAN777	61		56	
		MAN778	74		71	
Light chain	Protomer P1	Protein	201		176	
		Glycan 156				
		NAG718	17		16	
	Protomer P2	Protein	165		140	
Total surface area			1439		1298	
		by glycan	655	45.5%	573	44.1%

B. Hydrogen bonds and salt bridges between J038 and HIV-1 Env

		Protomer 1	Distance [Å]	Antibody
Heavy chain	1	F:LYS 171 [N]	3.72	X:PHE 100A [O]
	2	F:LYS 171 [NZ]	3.74	X:ASP 99 [O] *
	3	F:TYR 173 [OH]	2.75	X:ASP 100 [O]
	4	F:ARG 166 [O]	3.53	X:ARG 54 [NH2]
	5	F:ASP 167 [OD1]	2.76	X:TYR 33 [OH]
	6	F:LYS 169 [O]	2.96	X:TYR 100C [N]
	7	F:NAG 771 [O7]	3.62	X:TYR 100D [OH]
	8	F:NAG 771 [N2]	3.34	X:TYR 100D [OH]
	9	F:BMA 772 [O2]	3.73	X:ARG 54 [NH2]
	10	F:BMA 772 [O5]	3.61	X:ARG 54 [NH1]
	11	F:MAN 777 [O2]	2.91	X:ASN 30 [ND2]
Light chain	12	F:LYS 168 [NZ]	3.52	Y:TYR 91 [O]
	13	F:LYS 168 [NZ]	3.76	Y:ARG 92 [O]
	14	F:GLU 164 [OE1]	3.88	Y:ARG 93 [NH1]
	15	G:ASN 186 [ND2]	2.58	Y:ASP 1 [OD2]
Salt bridges				
		Protomer 1	Distance [Å]	Antibody
Light chain	1	F:GLU 164 [OE1]	3.88	Y:ARG 93 [NH1]

* UCA residues

Supplementary Table 5. Contributions of J038 and intermediate antibodies to binding of HIV-1 Env.

A. The interaction between J038 paratope residues and C1080 Env protein and glycans

				Buried surface area (Å²)									
				Protein	Glycan N156		Glycan N160						
	Residue	Amino acid	Hydrogen bond and salt bridge		glycan 717	718	770	771	772	776	777	778	Total per residue
Heavy chain	28	X:ALA28									3.4		3.4
	30	X:ASN30							16.3	29.5	35.4		81.2
	31	X:ASP31						16.6	4.5	1.2			22.3
	33	X:TYR33	H	39.0									39.0
	50	X:ARG50	H	36.3									36.3
	52	X:SER52		1.5									1.5
	54	X:ARG54		6.0				27.3	41.6	35.1		62.2	172.2
	55	X:ASP55		2.0								7.0	9.0
	57	X:TYR57		33.9									33.9
	59	X:GLU59		16.6									16.6
	76	X:TRP76								33.0	17.2	2.2	52.4
	101	X:PRO101						7.5					7.5
	102	X:GLU102						5.1					5.1
	103	X:ASP103	H	10.9									10.9
	104	X:ASP104	H	38.4	25.5	25.1							89.0
	105	X:PHE105	H	27.1		45.1							72.2
	106	X:GLY106		23.4									23.4
	107	X:TYR107	H	80.6			57.1	23.1					160.8
	108	X:TYR108		47.4			18.4	19.8					85.6
	109	X:GLN109		17.3									17.3
	110	X:PRO110		28.6									28.6
Light chain	1	Y:ASP1	H	25.8									25.8
	26	Y:THR26		6.3									6.3
	27	Y:GLN27		42.3									42.3
	28	Y:GLY28		20.4									20.4
	32	Y:ASP32		8.74									8.7
	52	Y:PHE52				15.9							15.9
	91	Y:TYR91	H	21.9									21.9
	92	Y:ARG92	H	58.3									58.3
	93	Y:ARG93	H, S	87.3									87.3
	94	Y:LEU94		36.9									36.9
	96	Y:LEU96		7.5									7.5

UCA residue

B. Increased binding surface areas to glycan by mutations accumulated on intermediate antibodies

Paratope	Heavy chain (Å²)	Light chain (Å²)	Increasing interface (Å²)	
Contribution by UCA	497.6	129.0	626.6	48%
By mutations in IA5	237.4	96.0	333.4	26%
By mutations in IA3	144.6	22.2	166.7	13%
By mutations in IA2	7.5	87.3	94.8	7%
By mutations in IA1	0	0	0	0%
Total	968.2	331.3	1299.5	
